# Supplementary material for: Owner-Reported Health Events in Australian Farm Working Dogs
Source: Animals (Basel). 2024 Jun 27;14(13):1895. doi: 10.3390/ani14131895 (PMC11240686; doi:10.3390/ani14131895)
Supplement: Supplementary file 1 [file animals-14-01895-s001.zip › animals-3075267-supplementary.pdf]

Identification number:

Australian Working Farm Dog Health Survey

June 2015

Researcher: Nicola Pattison BVSc
Phone: 0416784040, email: nic_pattison@hotmail.com

This project has been evaluated by peer review and judged to be low risk. Consequently, it has not been reviewed by one of the University's Human Ethics Committees. The researcher(s) named above are responsible for the ethical conduct of this research.

If you have any concerns about the conduct of this research that you wish to raise with someone other than the researcher(s), please contact Dr Brian Finch, Director (Research Ethics),

telephone 0011 64 6 356 9099, extn 86015, email: humanethics@massey.ac.nz.

**Owner/Handler Information**				
Age:	☐ < 20 years	☐ 21-30 years	☐ 31-40 years	☐ 41-50 years
	☐ 51-60 years	☐ 61-70 years	☐ > 70 years	
Gender:	☐ Male	☐ Female		
Job title:<br>(Tick all that apply)	☐ Owner	☐ Manager	☐ Employee	

**Property:**								
State:	☐ NSW	☐ VIC	☐ QLD	☐ ACT	☐ SA	☐ WA	☐ NT	☐ TAS
Property size in hectares:								

Number of working dogs over the age of 12 months on your property and/or with you here today: ....

Preventative health care:

**Frequency of vaccination:**	☐ Only as a pup	☐ Sporadically	☐ Once a year	☐ Never
	☐ Every .... year(s)		☐ Don't know	
**Frequency of worming:**	☐ Only as a pup	☐ Sporadically	☐ Once a year	☐ Never
	☐ Every .... month(s)		☐ Don't know	
**External parasite control:**	☐ Flea	☐ Mange mite		
How often?	☐ Sporadically (as needed)	☐ Every........... month(s)	☐ Don't know	

Does every dog on your farm receive the same treatment as indicated above? ☐ Yes ☐ No

Identification number:

For each working dog on your farm or here TODAY, please answer the questions below (Please do not use stud name/prefix):

Name of Dog: …………………………………………………………………………… **Age:** ……………………………………………………………

Breed: ……………………………………………………………………………………… **Sex:** ☐ Male ☐ Female

Is this dog used for breeding? ☐ Yes ☐ No

If YES, how many litters/matings has this dog had? ……………………………………………………………………………………

Neutered/desexed: ☐ Yes ☐ No

If YES, for what reason was this dog was neutered/desexed? ……………………………………………………………………………

What is the <u>main</u> use for this dog? ……………………………………………………………………………………………………

Is this dog registered with Local Shire/Council? ☐ Yes ☐ No **Insured?** ☐ Yes ☐ No

Type of stock worked/guarded: ☐ Sheep ☐ Beef cattle ☐ Dairy cattle ☐ Deer

(Tick all that apply) ☐ Goats ☐ Other (please specify): ……………………………………………………

Type of work: ☐ Livestock ☐ Paddock ☐ Yard ☐ Dog Trial

(Tick all that apply) ☐ Hunt ☐ Stock guarding ☐ Other: ……………………………………………………

IF dog competes at trials: ☐ Casual/Social ☐ Competitive (to accumulate points)

In the <u>last 12 months</u> has this dog been in work? ☐ Yes ☐ No

If YES, continue with the questions below for this dog.

If NO, please provide details (reason not in work): ……………………………………………………………………………………

……

Has this dog suffered any accidents or traumatic injury <u>in the last 12 months</u>? ☐ Yes ☐ No

If YES:

Type of injury:

- ☐ Puncture wound/Penetrating injury
- ☐ Broken bone
- ☐ Internal injury(ies)
- ☐ Laceration/Graze
- ☐ Joint dislocation/damage
- ☐ Tendon/Ligament damage
- ☐ Burn
- ☐ Don't know
- ☐ Other (details): ……………………………………………………………………………………………

Body part primarily affected:

☐ Hindleg	☐ Foreleg	☐ Paw	☐ Abdomen	☐ Chest
☐ Hip	☐ Shoulder	☐ Head	☐ Mouth/Teeth	☐ Eye
☐ Ear	☐ Spinal	☐ Tail	☐ Multiple areas	☐ Other

Details: ……

What caused it? ☐ Injury by stock ☐ Motor vehicle injury ☐ Caught in fence
☐ Dog fight/bite ☐ Fall (not vehicle related) ☐ Gunshot
☐ Caught in chain ☐ Injured by wildlife ☐ Unknown
☐ Other (specify): ……………………………………………………………………………

Details: ……………………………………………………………………………………………

Did this dog receive veterinary treatment for its injury(ies)? ☐ Yes ☐ No

If YES: ☐ Surgery ☐ Hospitalisation ☐ Medication ☐ Other (describe): ……………………

Details: ……………………………………………………………………………………………

Apart from accident or injury, <u>in the last 12 months,</u> have you noticed any illness/disease or any health problems in this dog? ☐ Yes ☐ No

If YES, please tick one or more boxes below, indicate the details of the problem, and whether or not the dog received veterinary attention for the problem:

	Seen by vet	Details
☐ Allergy/Autoimmune Disease (1)	☐ Yes ☐ No	
☐ Cancer/Tumour (2)	☐ Yes ☐ No	
☐ Degenerative/Old Age Problem (3)	☐ Yes ☐ No	
☐ Dental/Oral Disease or Problem (4)	☐ Yes ☐ No	
☐ Ear/Eye Disease or Problem (5)	☐ Yes ☐ No	
☐ External/Internal Parasitism (6)	☐ Yes ☐ No	
☐ Gastrointestinal/Abdominal Disease or Problem (7)	☐ Yes ☐ No	
☐ Genetic/Inherited Disease or Problem (8)	☐ Yes ☐ No	
☐ Heat Stroke (9)	☐ Yes ☐ No	
☐ Heart/Lung/Airway Disease (10)	☐ Yes ☐ No	
☐ Infection/Abscess (11)	☐ Yes ☐ No	
☐ Leg/Paw Problem (not related to trauma) (12)	☐ Yes ☐ No	
☐ Poisoning (13)	☐ Yes ☐ No	
☐ Reproductive/Urinary Tract Disease (14)	☐ Yes ☐ No	
☐ Skin/Coat Disease or Problem (15)	☐ Yes ☐ No	
☐ Snake/Insect/Spider Bite (16)	☐ Yes ☐ No	
☐ Spinal/Neurological Disease (17)	☐ Yes ☐ No	
☐ Whelping problem/pregnancy issue (18)	☐ Yes ☐ No	

☐ Other Problem: Seen by vet: ☐ Yes ☐ No

Describe: ……………………………………………………………………………………………

Has the above illness or injury caused this dog to miss days it would normally work? ☐ Yes ☐ No

If YES, what period of restricted exercise/cage rest did this dog require? ……………………………

Has this dog returned to work in its previous capacity since the illness/injury? ☐ Yes ☐ No

If NO, what is this dog doing now? ☐ Retired from work ☐ Still recovering ☐ Working, but in a reduced capacity (semi-retired)

Thank you for participating in this survey today.

Identification Number:

For Breeders or Trainers of working dogs: Do you usually sell trained or started working dogs over the age of 12 months? ☐ Yes ☐ No
If yes, how many dogs over the age of 12 months have you sold as working dogs in the last year? ………..

Please answer the questions below for dogs <u>over the age of 12 months</u> that have been on your property in the last 12 months and are no longer on your property or here with you TODAY (please <u>do not</u> use stud names):

Dog (name):	Age:	Breed:	Sex:	Desexed:	Reason dog no longer on your property TODAY?
1				☐ Yes ☐ No	☐ Died ☐ Sold as working dog ☐ Other reason: ………………………………..
2				☐ Yes ☐ No	☐ Died ☐ Sold as working dog ☐ Other reason: ………………………………..
3				☐ Yes ☐ No	☐ Died ☐ Sold as working dog ☐ Other reason: ………………………………..
4				☐ Yes ☐ No	☐ Died ☐ Sold as working dog ☐ Other reason: ………………………………..

If the dog died, did it die naturally, or was it euthanased?

Dog (name):	Died naturally (tick)	Euthanased by: (circle)	Reason that the dog died or was euthanased?	Details:
1		Vet / On farm	☐ Illness ☐ Injury ☐ Old age ☐ Lack of working instinct/behaviour	
2		Vet / On farm	☐ Illness ☐ Injury ☐ Old age ☐ Lack of working instinct/behaviour	
3		Vet / On farm	☐ Illness ☐ Injury ☐ Old age ☐ Lack of working instinct/behaviour	
4		Vet / On farm	☐ Illness ☐ Injury ☐ Old age ☐ Lack of working instinct/behaviour	

Thank you for participating in this survey today.
